# A Metabolomics Exploration of Young Lotus Seeds Using Matrix-Assisted Laser Desorption/Ionization Mass Spectrometry Imaging

**DOI:** 10.3390/molecules30153242

**Published:** 2025-08-01

**Authors:** Ying Chen, Xiaomeng Xu, Chunping Tang

**Affiliations:** 1 University of Chinese Academy of Sciences, Beijing 100049, China; chenying1@simm.ac.cn; 2 State Key Laboratory of Drug Research, Shanghai Institute of Materia Medica, Chinese Academy of Sciences, Shanghai 201203, China; 3 School of Pharmacy, Henan University, Kaifeng 475004, China; xuxiaomeng@simm.ac.cn

**Keywords:** louts seed, plumule, MALDI-MSI, secondary metabolites, spatial distribution

## Abstract

Lotus (*Nelumbo nucifera* Gaertn.) is a quintessential medicinal and edible plant, exhibiting marked differences in therapeutic effects among its various parts. The lotus seed constitutes a key component of this plant. Notably, the entire seed and the plumule display distinct medicinal properties. To investigate the “homologous plants with different effects” phenomenon in traditional Chinese medicine, this study established a Matrix-Assisted Laser Desorption/Ionization Mass Spectrometry Imaging (MALDI-MSI) method. This study employed immature lotus seeds as the experimental material, diverging from the mature seeds conventionally used. Conductive double-sided tape was employed for sample preparation, and complete longitudinal sections of the seeds were obtained, followed by MALDI-MSI analysis to identify and visualize the spatial distribution of characteristic secondary metabolites within the entire seeds. The results unveiled the diversity of metabolites in lotus seeds and their differential distribution across tissues, with pronounced distinctions in the plumule. A total of 152 metabolites spanning 13 categories were identified in lotus seeds, with 134, 89, 51, and 98 metabolites discerned in the pericarp, seed coat, cotyledon, and plumule, respectively. Strikingly, young lotus seeds were devoid of liensinine/isoliensinine and neferine, the dominant alkaloids of mature lotus seed plumule, revealing an early-stage alkaloid profile that sharply contrasts with the well-documented abundance found in mature seeds and has rarely been reported. We further propose a biosynthetic pathway to explain the presence of the detected benzylisoquinoline and the absence of the undetected bisbenzylisoquinoline alkaloids in this study. These findings present the first comprehensive metabolic atlas of immature lotus seeds, systematically exposing the pronounced chemical divergence from their mature counterparts, and thus lays a metabolomic foundation for dissecting the spatiotemporal mechanisms underlying the nutritional and medicinal value of lotus seeds.

## 1. Introduction

Medicinal plants are gaining global attention for their health benefits [1]. The spatial distribution of bioactive compounds in these plants is crucial but often lost during the extraction and identification using traditional phytochemical methods [2]. Matrix-Assisted Laser Desorption/Ionization Mass Spectrometry Imaging (MALDI-MSI) offers a label-free solution for in situ compound localization with high spatial resolution, making it ideal for small molecule analysis [3]. Recently, MALDI-MSI has been widely applied in plant metabolomics, providing crucial support for understanding bioactive compound distribution, biosynthetic pathways, plant variety identification, growth stage determination, and plant–ecosystem interactions [4,5,6,7,8,9,10].

In traditional Chinese medicine, the phenomenon of “homologous plants with different effects” is common, where different parts of the same medicinal plant exhibit distinct therapeutic properties due to varying distributions of bioactive secondary metabolites [11]. For example, *Nelumbo nucifera* Gaertn., a typical medicinal and edible plant, exhibits notable functional differentiation among its parts [12]. Its seeds, known as lotus seeds, rich in essential nutrients (lipids, proteins, starch, etc.) and bioactive compounds [13,14,15], are known for their spleen-strengthening, diarrhea-relieving, and heart-calming effects. In contrast, the plumule, the green embryo or core of the lotus seed, is recognized for its heat-clearing, detoxifying, and sedative properties [16].

The green plumule of the mature lotus seed, known as *Nelumbinis plumula*, is officially documented in the Pharmacopoeia of the People’s Republic of China. Benzylisoquinoline alkaloids, the most notable liensinine, isoliensinine, and neferine, have been identified as the principal bioactive constituents responsible for its effects [17]. Current pharmacopoeial standards stipulate that qualified lotus plumule must contain not less than 0.2% liensinine (2015 edition) or not less than 0.7% neferine (2020 edition, calculated on a dry-weight basis).

The high alkaloid content of the lotus plumule imparts an intense bitterness; however, the young lotus seeds consumed as food are mildly sweet and devoid of this bitterness. This suggests that the plumule in young seeds either lacks these alkaloids or contains them at negligible levels. In other words, despite extensive research on mature or unspecified lotus seeds, the metabolic profile of young seeds diverges markedly from that of their mature counterparts.

In this study, immature rather than mature lotus seeds were selected as the experimental material, and MALDI-MSI technology was employed to analyze the entire lotus seeds, providing vivid insights into tissue heterogeneity and the spatial distribution of bioactive compounds within the seed. These findings may help elucidate the relationship between bioactive compound distribution and the pharmacological properties of lotus seeds, contributing to a deeper exploration of their nutritional and medicinal value and offering valuable clues for deciphering the biosynthetic pathways of bioactive compounds in lotus seeds.

## 2. Results

### 2.1. Structural Organization of Fresh Lotus Seed

**Figure 1:**
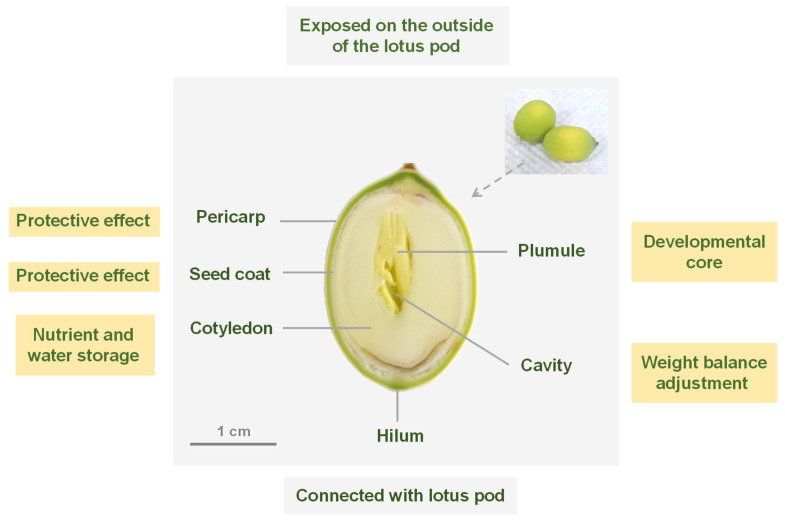
Anatomical organization of fresh lotus seed as revealed by frozen section imaging.

The fresh lotus seed could be anatomically divided into the pericarp, seed coat, cotyledon, and plumule, progressing from the outermost layer to the innermost layer, as illustrated in Figure 1 through the optical imaging of its frozen section. The lotus seed was observed to exhibit an anatropous ovule structure, where it was attached to the seedpod through its lower concave region, while the upper convex part protruded outside the seedpod. The yellow-green pericarp and the milky-white seed coat were identified as protective barriers, offering mechanical protection and preventing moisture loss. The cotyledons were found to be reservoirs rich in proteins, starch, and lipids, essential for storing water and nutrients. The plumule was determined to constitute the developmental nucleus of the lotus seed. A cavity was observed separating the plumule from the cotyledons, aiding in regulating the seed’s weight distribution and ensuring its stable morphology during the maturation process.

### 2.2. Preparation of Frozen Section of Fresh Lotus Seed

Obtaining intact and non-overlapping lotus seed sections through direct slicing is challenging due to the complex tissue structure of lotus seed and the cavity between the cotyledons and the plumule. Kawamoto et al. introduced a technique for preparing fresh-frozen sections from hard tissues, whole animals, insects, and plants using adhesive films [18]. Similarly, Zaima et al. used the adhesive films to obtain rice sections [19]. Shin Hye Kim et al. successfully obtained the whole corn seed sections using a different type of tape [20].

**Figure 2:**
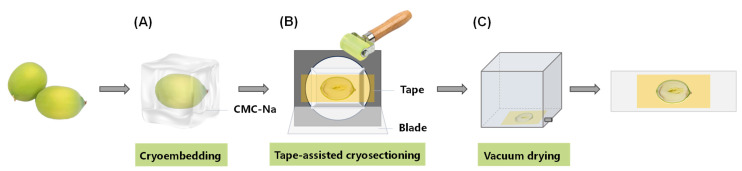
Schematic diagram of sample preparation process. (**A**) Cryoembedding: Isopentane was pre-cooled in dry ice. Lotus seeds were extracted from the pod and promptly immersed in a solution of CMC–Na within an aluminum foil container, which was then submerged in the pre-cooled isopentane for rapid freezing. (**B**) Cryosectioning aided by conductive double-sided tape: The cryostat temperature was maintained at −20 °C. As the lotus seed tissue was gradually trimmed to the desired position, it was affixed onto the tape and subsequently sectioned. (**C**) Vacuum drying: The tape containing the lotus seed slices was quickly transferred to a vacuum-drying oven for drying before being mounted onto an ITO conductive glass slide.

In this study, lotus seeds were processed by frozen sectioning, with the sections fixed using conductive double-sided tape, as illustrated in Figure 2. Upon removal from the lotus pod, the fresh seeds were promptly embedded in carboxymethylcellulose sodium (CMC-Na) and sliced at −20 °C using a cryostat. The tape was affixed to the tissue once the desired position was reached, enabling the acquisition of intact lotus seed slices on the tape. Subsequently, vacuum drying was performed, and the tape was then secured onto an indium tin oxide (ITO) glass slide to finalize sample preparation. This procedure yielded structurally preserved, undistorted lotus seed sections devoid of overlap, facilitating the generation of high-fidelity mass spectrometry imaging data. Furthermore, it served as a valuable protocol for handling similarly intricate plant specimens that pose challenges for direct sectioning.

It is noteworthy that variations in the orientation of lotus seed placement during embedding and sectioning may result in discontinuities in the seed core tissue. However, these discrepancies can be mitigated through careful examination of the sections and analysis of metabolite distribution patterns.

### 2.3. Metabolite Identification and Statistical Analysis of Metabolite Distribution

#### 2.3.1. Establishment of In-House Compound Library for Lotus and Lotus Seed

The natural product source module of the Reaxys database (https://www.reaxys.com/#/search/advanced (accessed on 27 November 2024) was utilized to search for compounds isolated from lotus seeds using the keyword “*Nelumbo nucifera*”. As of 27 November 2024, 241 entries were retrieved. Additionally, 87 compounds isolated from lotus seeds were gathered from literature sources, predominantly review articles and Chinese publications [11,12,21]. By consolidating these datasets and eliminating duplicates, an exclusive in-house compound library for the identification of secondary metabolites in lotus seeds was established, comprising 277 compounds across 15 metabolite classes, such as alkaloids, flavonoids, apocarotenoids, phenolic acids, fatty acids, phenylpropanoids, and others (Table S1 in Supplementary Materials). Metabolite classification was performed using NPClassifier (https://github.com/mwang87/NP-Classifier (accessed on 17 December 2024) based on their SMILES strings [22]. This library enables a more precise identification of secondary metabolites in lotus seeds through comparison with the accurate molecular masses of compounds detected via MALDI-MSI.

#### 2.3.2. Statistical Analysis of the Distribution of Secondary Metabolites in Lotus Seeds

After normalization of the raw data, the 1020 peaks with the highest response intensity were processed. Imported into the in-house compound library, metabolites were identified as molecular ions according to their unique *m*/*z* values, including [M + H]^+^, [M + Na]^+^, [M − H]^−^, [M + Cl]^−^, and other specific adducts.

**Figure 3:**
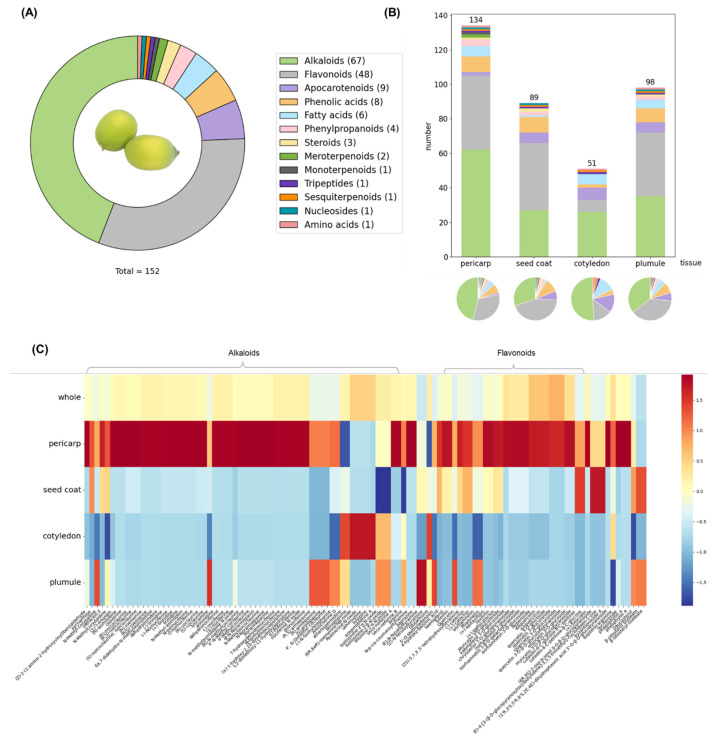
Metabolite distribution statistics in lotus seeds. (**A**) Circular diagram showing the distribution of 13 metabolite classes in the entire lotus seed. (**B**) Stacked bar chart and pie chart illustrating the distribution of 13 metabolite classes in different lotus seed tissues (pericarp, seed coat, cotyledons, and plumule), with a shared legend used across the circular diagram. (**C**) Heatmap analysis depicting the richness of metabolite composition in lotus seeds. The horizontal axis listed metabolite names according to their *m*/*z* values and categories, while the vertical axis represented the different lotus seed tissues. Expression levels were represented by a color gradient, ranging from deep blue (low) to yellow (medium) and deep red (high).

Compounds exhibiting poor peak shape or evident distribution in the blank control were excluded from the analysis. Ultimately, 152 compounds were identified (Table S2 in Supplementary Materials), comprising 67 alkaloids, 48 flavonoids, 9 apocarotenoids, 8 phenolic acids, 6 fatty acids, 4 phenylpropanoids, and 10 other compounds, including meroterpenoids, as illustrated in Figure 3A. Notably, significant challenges remain in identifying isomers via mass spectrometry imaging [23], leading to the separate listing of some compounds and their isomers.

To examine the distribution of secondary metabolite categories across lotus seed tissues, we conducted a detailed data analysis, with findings shown in Figure 3B. We identified 134 metabolites in the pericarp, 89 in the seed coat, 51 in the cotyledons, and 98 in the plumule. The pie chart clearly depicts the proportions of compound types within each tissue, highlighting alkaloids (green) and flavonoids (gray) as predominant across all tissues. The cotyledon exhibits the highest proportion of alkaloids, slightly exceeding that of the pericarp and significantly surpassing both the seed coat and plumule. Conversely, the seed coat and plumule display a relatively higher proportion of flavonoids compared to the cotyledon. The stacked bar chart provides an intuitive comparison of metabolite quantity and diversity among the tissues.

Figure 3C depicts a heatmap of the relative abundance obtained from semi-quantitative analysis of diverse secondary metabolites across various tissues of the lotus seed. Consistent with previous reports [24,25], alkaloids and flavonoids were identified as key bioactive components in lotus seeds, with widespread distribution throughout the seed. Notably, the pericarp exhibited the greatest metabolic diversity, harboring the highest number and abundance of alkaloids and flavonoids. However, when lotus seeds are consumed or used for medicinal purposes, the pericarps are typically discarded.

Significant metabolic diversity is also evident in the plumule and seed coat. Previous studies have shown that alkaloid and flavonoid levels in the plumule increase considerably as lotus seeds mature [26]. In contrast, this study utilized immature lotus seeds, which had not yet accumulated substantial levels of these compounds, especially alkaloids, which might account for the experimental results obtained.

It should be noted that the distribution of the metabolite types depicted in Figure 3A,C is based on their number from the total number of identified compounds. As a matter of fact, although a great number of alkaloids have been identified, their total content is less than that of flavonoids. The result is consistent with that reported in literature [11].

### 2.4. Identification and Localization of Characteristic Metabolites in Lotus Seeds

#### 2.4.1. Characteristic Metabolites Identified Under Positive Ion Mode of MALDI-MSI Detection

**Figure 4:**
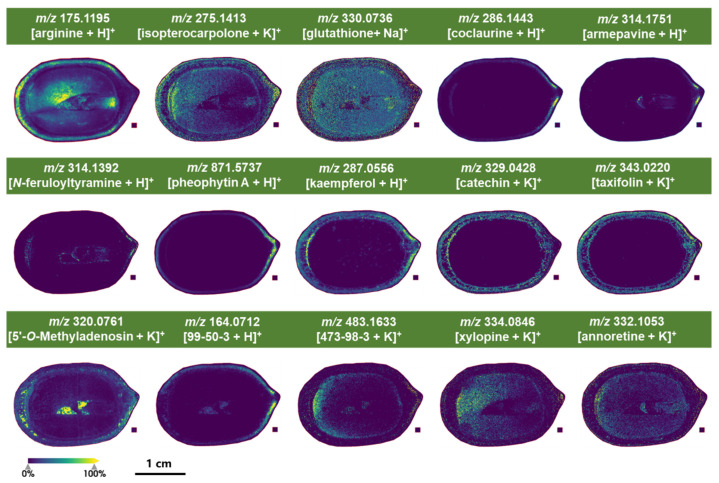
Partial characteristic secondary metabolites of lotus seeds identified under positive ion mode detection. The metabolites were arranged according to their distribution patterns within the tissues. Due to the long names of some metabolites, their CAS numbers were indicated in the figure for clarity. 99-50-3: (*Z*)-2-(1-amino-2-hydroxyvinyl) benzaldehyde, 473-98-3: (1′*R*,3′*S*,5′*R*,8′*S*,2*E*,4*E*)-dihydrophaseic acid 3′-*O*-*β*-D-glucopyranoside.

MALDI mass spectrometry imaging was conducted in both positive and negative ion modes. α-Cyano-4-hydroxycinnamic acid (CHCA) is one of the most widely used matrices for MALDI-MS imaging of small molecules [27]. It is suitable for the detection of various metabolites, such as alkaloids, lipids, proteins, and peptides [28,29,30]. In this study, CHCA served as the matrix for positive ion detection, as it has been described in the literature to form small crystals, producing a more homogeneous matrix coating [31]. The findings revealed distinct differences in metabolite distribution within young lotus seeds. Metabolites with higher response intensity or unique distribution were identified as representative. Figure 4 illustrates these metabolites based on their tissue distribution patterns.

Arginine (*m*/*z* 175.1195) is a key component of plant nitrogen metabolism, playing a crucial role in growth, development, and formation of resistance mechanisms [32]. This amino acid is distributed throughout the pericarp, cotyledon, and hypocotyl of lotus seeds.

Glutathione (*m*/*z* 330.0736) is involved in nutrient metabolism, antioxidant defense, and regulation of cellular metabolic functions ranging from gene expression, DNA and protein synthesis to signal transduction, cell proliferation, and apoptosis [33]. This tripeptide is widely distributed throughout the lotus seeds.

Isopterocarpolone (*m*/*z* 275.1413), a sesquiterpene first isolated from lotus leaves by Chen et al. [34], exhibits lipid-lowering activity and is also widely distributed in lotus seeds.

The benzylisoquinoline alkaloids coclaurine (*m*/*z* 286.1443) and armepavine (*m*/*z* 314.1751) are also present, with armepavine potentially derived from coclaurine through *N*-methylation and *O*-methylation steps. Coclaurine is primarily localized at the apex of the lotus seed pericarp, while armepavine is found not only in the pericarp apex but also in the plumule. This distribution pattern may be related to the specific localization of enzymes catalyzing methylation reactions within the lotus seed.

*N*-feruloyltyramine (*m*/*z* 314.1392) exhibits antioxidant activity and plays roles in wound healing and defense responses in plants [35,36]. It is specifically distributed at the apex of the pericarp, the base of the seed coat, and within the plumule.

Additionally, two alkaloids, xylopine (*m*/*z* 334.0846) and annoretine (*m*/*z* 332.1053), were localized within the cotyledons and the plumule.

Pheophytin A (*m*/*z* 871.5737), a magnesium-free degradation product of chlorophyll during photosynthesis, was specifically localized at the apex of the lotus seed pericarp, corresponding to the part of the fresh seed exposed to sunlight outside the seedpod.

Flavonoids, key pharmacologically active compounds in lotus seeds, serve vital plant functions, including antioxidant protection, pest and disease defense, UV resistance, and metabolic regulation [37,38].

Kaempferol (*m*/*z* 287.0556), catechin (*m*/*z* 329.0428), and taxifolin (*m*/*z* 343.0220) are localized in the pericarp and seed coat. Catechin is synthesized through phenylpropanoid biosynthetic pathway by phenylalanine ammonia-lyase (PAL), cinnamate 4-hydroxylase (C4H), and 4-Coumarate: CoA ligase (4CL) [39]. It could help to reduce reactive oxygen species and improve the environmental adaptability of plants [40].

5′-*O*-methyladenosine (*m*/*z* 320.0761), a nucleoside involved in plant metabolism and signal transduction, is present in the pericarp, seed coat, and cavity between the cotyledons and plumule.

The lotus seed metabolome exhibited distinct spatial distribution patterns and high response intensities for several unidentified metabolites (Figure S1 in Supplementary Materials). These uncharacterized compounds may represent previously unexplored chemical constituents within the lotus seed, warranting further in-depth metabolomic investigation.

#### 2.4.2. Characteristic Metabolites Identified Under Negative Ion Mode of MALDI-MSI Detection

**Figure 5:**
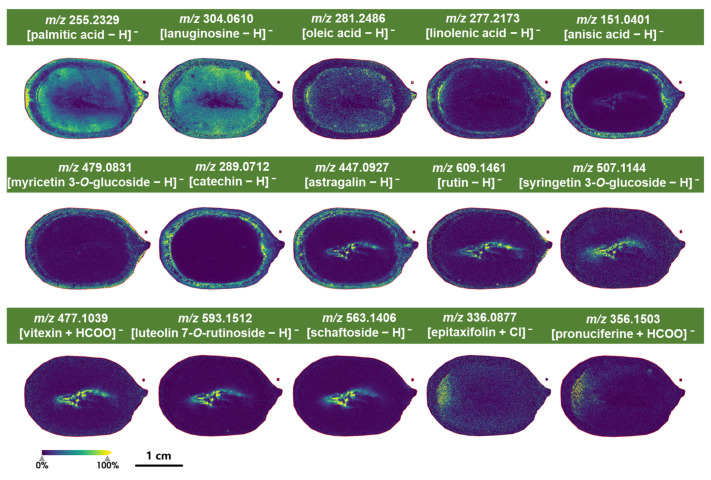
Partial characteristic secondary metabolites of lotus seeds identified under negative ion mode detection.

A commonly used matrix 9-aminoacridine (9AA) was chosen for detection in negative ion mode. It is suitable for detecting various types of metabolites, such as organic acids, phenolic acids, lipids, and glucoside [41,42,43]. Metabolites with high-response intensity or specific distribution were selected as representatives. Results indicated that fatty acids and organic acids in lotus seeds showed higher response intensities in this mode. Their distribution patterns within tissues are illustrated in Figure 5.

Palmitic acid (*m*/*z* 255.2329), a cell membrane component involved in energy storage and plant growth regulation [44], was found in the pericarp, cotyledons, and plumule of lotus seeds.

Oleic acid (*m*/*z* 281.2486), also a membrane component linked to energy storage, was localized in the cotyledons and hypocotyl.

Linolenic acid (*m*/*z* 277.2173), which plays a role in plant signal transduction and stress responses, was predominantly located in the pericarp and seed coat, mirroring the distribution of anisic acid (*m*/*z* 151.0401).

Flavonoids were also effectively detected in negative ion mode. Myricetin 3-*O*-glucoside (*m*/*z* 479.0831) and catechin (*m*/*z* 289.0712) were specifically located in the pericarp and seed coat of lotus seeds, likely serving as defenses against pests and UV radiation.

Astragalin (*m*/*z* 447.0927) was found in the pericarp, seed coat, plumule, and the cavity between the plumule and cotyledons.

Rutin (*m*/*z* 609.1461) appeared in the pericarp, plumule, and cavity between the plumule and cotyledons.

Syringetin 3-*O*-glucoside (*m*/*z* 507.1144), vitexin (*m*/*z* 477.1039), luteolin 7-*O*-rutinoside (*m*/*z* 593.1512), and schaftoside (*m*/*z* 563.1406) were localized in the plumule and its cavity with the cotyledons.

Epitaxifolin (*m*/*z* 336.0877) was distributed in the cotyledons, particularly at the base where they connect with the seed coat.

A few alkaloids were also detected in negative ion mode. Lanuginosine (*m*/*z* 304.0610) was widespread throughout the seed, except in the internal cavity, while pronuciferine (*m*/*z* 356.1503) shared a similar distribution pattern with epitaxifolin. Pronuciferine is a naturally occurring proaporphine alkaloid that belongs to isoquinoline alkaloids, which possesses a 4′-substituted benzyl group, implying the presence of a novel uncharacterized dehydration reaction involved in the conversion of pronuciferine to nuciferine [45].

It should be pointed out that combining positive and negative ion detection modes provided more comprehensive information on the specific distribution of metabolites within lotus seeds.

## 3. Discussion

In this study, we aimed to identify bioactive compounds in young lotus seeds, with a particular emphasis on chemical profiling. As a result, it is surprising to find that liensinine/isoliensinine and neferine, three bis-benzylisoquinoline alkaloids renowned for their significant pharmacological activities and recognized as representative compounds in lotus plumule [46,47,48], were absent from our results. We have also confirmed this hypothesis by extracting the lotus seeds with methanol and analyzing the extract by LC-MS/MS.

We propose that the immaturity of the lotus seeds used in this study might be a key factor contributing to the undetection of these compounds. This is supported by observations of the pericarp color and the development stage of the plumule [49]. Previous research has also shown that the expression of genes related to benzylisoquinoline alkaloid biosynthesis is very low in the early stages of lotus seed development, making alkaloid levels in the plumule undetectable at this stage. As the seeds mature, the expression of these genes increases gradually, leading to alkaloid accumulation [11,50]. This explains the low distribution of benzylisoquinoline alkaloids in the plumule.

**Figure 6:**
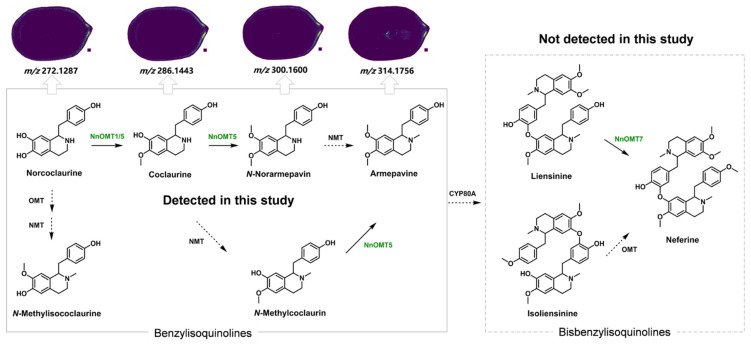
Proposed biosynthetic pathways of the benzylisoquinoline alkaloids detected by the MALDI-MSI analysis (left solid box) and the undetected bisbenzylisoquinoline alkaloids (right dashed box). The enzymes catalyzing the corresponding conversions were indicated near the arrows. Enzymes that have been identified and verified in lotus are denoted by green text, and reactions are indicated by solid black arrows; enzymes that have not been verified but are inferred from literature are represented by black text, with dashed black arrows used to indicate the reactions.

The MALDI-MSI results also shed light on enzymes related to benzylisoquinoline alkaloid formation. The distribution of benzylisoquinoline alkaloids, including norcoclaurine (*m*/*z* 272.1287), coclaurine (*m*/*z* 286.1443), *N*-norarmepavine (*m*/*z* 300.1600), *N*-methylisococlaurine (*m*/*z* 300.1600), *N*-methylcoclaurin (*m*/*z* 300.1600), and armepavine (*m*/*z* 314.1751), in the pericarp and embryo suggests that relevant enzymes, e.g., NnOMT1 and NnOMT5 [51], are concentrated in the pericarp, with traces possibly migrating from the pericarp to the plumule at the studied development stage. At this very young stage, CYP80A subfamily enzymes [52], which catalyze the formation of the core structure of bis-benzylisoquinoline alkaloids through an intermolecular C-O phenolic coupling of two benzylisoquinoline alkaloid monomers, are likely present in very low concentrations or are even absent in the plumule, which might explain the absence of liensinine/isoliensinine and neferine (Figure 6).

Additionally, it should be noted that this study has certain limitations. First, MALDI-MSI inherently lacks the capability to distinguish structural isomers. While constructing an in-house compound library improved identification accuracy, it does not fundamentally address the challenge of isomer discrimination. Incorporating MS/MS confirmation or orthogonal validation techniques (e.g., LC-MS/MS or the use of authentic standards) may help to enhance the reliability of isomer identification. Second, tissue heterogeneity and complex sample composition can cause matrix effects in MALDI-MSI, impacting ionization efficiency and signal accuracy. To mitigate this, we standardized sample preparation, ensured uniform matrix crystallization using an automated sprayer, and applied RMS normalization to reduce variability. These measures effectively minimized matrix effects and improved the reliability of spatial imaging data.

## 4. Materials and Methods

### 4.1. Plant Material and Reagents

The lotus pods were harvested 3–4 days after flower wilting and procured from Yongzhou, Hunan Province, China, in May 2024 and identified by Professor Jin-Gui Shen of Shanghai Institute of Materia Medica, Chinese Academy of Sciences. Upon acquiring the fresh lotus pods, the lotus seeds were extracted, frozen immediately, and embedded to quench metabolic processes.

α-Cyano-4-hydroxycinnamic acid (CHCA, CAS number: 28166-41-8) and 9-aminoacridine (9AA, CAS number: 90-45-9) were acquired from Sigma-Aldrich (St. Louis, MO, USA). Methanol (MeOH) and acetonitrile (ACN) of HPLC-grade were obtained from Merck (Rahway, NJ, USA). Ultrapure water was produced using the Millipore Milli-Q Advantage A10 purification system (Millipore, Molsheim, France). Indium tin oxide (ITO)-coated conductive glass slides were sourced from Bruker Daltonics (Bremen, Germany). Sodium carboxymethyl cellulose (CMC-Na) was obtained from Huai’an Shanghai Chemical Reagent Co., Ltd. (Huai’an, China).

### 4.2. Sample Preparation for MALDI-MSI Analysis

The tissues were immersed in a 2% CMC–Na solution within an embedding box, followed by rapid freezing in pre-cooled isopentane at −78.5 °C for 2 min to produce frozen CMC–Na blocks suitable for sectioning.

Frozen tissues were sliced into 12 μm sections using a Leica CM1950 cryostat (Leica Biosystems, Nussloch, Germany) at a chamber temperature of −20 °C. After obtaining the desired section, a piece of conductive double-sided tape (Tesa, Norderstedt, Germany) was evenly placed on the tissue. The tape, along with the attached lotus seed slice, was then transferred into a vacuum oven (Jiangsu Heqi Scientific Instrument Co., Ltd., Yancheng, China) at room temperature for 10 min to allow for moisture evaporation. Following the drying process, the opposite side of the tape was adhered to the ITO glass slide.

For positive-mode MALDI, a solution of 10 mg·mL^−1^ CHCA in a mixture of ACN–water (7:3, *v*/*v*) with 0.1% trifluoroacetic acid (TFA) was sprayed uniformly onto the sample substrate using an HTX TM-Sprayer (HTX technologies, Chapel Hill, NC, USA). For negative-mode MALDI, a solution of 10 mg·mL^−1^ 9AA in a mixture of methanol–water (9:1, *v*/*v*) was applied. Subsequently, the samples were prepared for data acquisition.

### 4.3. Data Acquisition by MALDI-MSI and Data Analysis

Data were acquired using a timsTOF fleX^TM^ mass spectrometer (Bruker Daltonics, Billerica, MA, USA), equipped with a 10 kHz SmartBeam 3D UV laser source (355 nm) for MALDI ionization in positive mode. The MALDI laser operated at a frequency of 10,000 Hz with 50% laser power, 400 accumulated laser shots, and data acquisition at a spatial resolution of 50 μm across the mass range of *m*/*z* 50-1200 Da. Instrument calibration was conducted using an Agilent tuning mix (Agilent Technologies, Santa Clara, CA, USA). Data analysis for MALDI-MS was performed using DataAnalysis (ver. 5.3, Bruker Daltonics, Bremen, Germany). The timsControl software (version 4.0.4, Bruker Daltonics, Germany) was employed to set the ion source and mass spectrometer parameters. Optical images of tissue sections were uploaded into Flex Imaging (version 7.1, Bruker Daltonics, Germany) to define the scan areas.

### 4.4. Raw Data Processing of MSI

Data visualization and correlation analysis were performed using SCiLS Lab software (version 2024a Pro, Bruker Daltonics, Germany), with normalization achieved through root mean square methodology.

### 4.5. Semi-Quantification Approach

To characterize spatial metabolic heterogeneity, K-means clustering was applied to the MSI dataset, segmenting the tissue into 10 distinct clusters (Figure S2 in Supplementary Materials). Based on the clustering results and the inherent structure of the lotus seeds, manual segmentation of the pericarp, seed coat, cotyledon, and plumule regions was performed using SCiLS Lab software. The ion intensities of metabolites within each tissue were calculated and exported as CSV files. Heatmaps were subsequently generated using Jupyter Notebook (version 7.0.8).

## 5. Conclusions

This study employed a sample preparation technique utilizing conductive double-sided tape to achieve intact lotus seed sections for analysis. A well-established MALDI-MSI method was utilized to detect and spatially map characteristic metabolites within young lotus seeds.

A total of 152 metabolites distributed across 13 chemical classes were annotated in young lotus seeds, with 134, 89, 51, and 98 of these being spatially resolved in pericarp, seed coat, cotyledon, and plumule, respectively. Alkaloids and flavonoids emerged as the two most abundant classes, both in terms of cumulative content and compound diversity, mirroring the metabolite hierarchy previously established for mature seeds.

The metabolomic profile of young lotus seeds reveals a striking divergence from that of mature seeds: liensinine, isoliensinine, and neferine—the principal, high-abundance alkaloids in mature lotus plumules—are absent in the young seeds, thereby accounting for their naturally sweet, non-bitter taste.

These outcomes underscore the substantial chemical diversity of lotus seed metabolites and their non-uniform distribution across distinct tissues. These findings not only elucidate the metabolic foundation governing tissue-specific specialization but also offer contemporary scientific validation for the concept of “homologous plants with different effects” in traditional Chinese medicine, thereby providing valuable insights into the nutritional and medicinal potential of lotus seeds.

Spatial distribution patterns identified through MALDI-MSI provide insights into the biosynthetic pathways of important plant metabolites. In this study, the absence of liensinine/isoliensinine and neferine in the plumule of young lotus seeds provides clues to elucidate the biosynthetic pathway of the important bisbenzylisoquinoline alkaloids. Nonetheless, a range of mono-benzylisoquinoline alkaloids exhibiting significant spatial heterogeneity were observed, indicating their likely participation in tissue-specific metabolic pathways during seed maturity process.

In the future, integrating MALDI-MSI data across the entire ripening stages of lotus seeds will be performed, enabling a dynamic monitoring of spatial and temporal alterations in essential metabolites. Leveraging this metabolomic atlas, the enzymes and regulatory factors that govern the designated biosynthetic steps will be characterized.

In addition, lotus seeds have been traditionally consumed for their cotyledons and plumule, with occasional use of the seed coat while the pericarp is typically discarded. However, recent attention has been drawn to the analysis of bioactive compounds in the pericarp, revealing its antioxidant properties, inhibition of pre-adipocyte differentiation [53], dose-dependent suppression of HePG2 cell proliferation [54], and cytoprotective effects [55]. The extract from lotus seed pericarp has found applications as an additive in Chinese Cantonese-style sausages and as a substrate for mushroom cultivation. Our findings in this study demonstrated a rich content of alkaloids and flavonoids in the pericarp of young lotus seeds, providing scientific evidence for its potential as a source of functional food or dietary supplement.

## Data Availability

The original contributions presented in this study are included in the article/Supplementary Materials. Further inquiries can be directed to the corresponding authors.

## Supplementary Materials

The following supporting information can be downloaded at: https://www.mdpi.com/article/10.3390/molecules30153242/s1, Figure S1: Some images of the unidentified metabolites under positive ion mode detection; Figure S2: The classification results of the MSI data (under positive ion mode detection) after K-means spatial clustering. Table S1: The in-house compound library of the lotus seed; Table S2: The list of identified metabolites.

## Abbreviations

The following abbreviations are used in this manuscript:

MALDI-MSI	Matrix-Assisted Laser Desorption/Ionization Mass Spectrometry Imaging
CMC-Na	Carboxymethylcellulose sodium
ITO	Indium–tin oxide
CHCA	α-Cyano-4-hydroxycinnamic acid
9AA	9-Aminoacridine
