# Supplementary material for: A Metabolomics Exploration of Young Lotus Seeds Using Matrix-Assisted Laser Desorption/Ionization Mass Spectrometry Imaging
Source: Molecules. 2025 Aug 1;30(15):3242. doi: 10.3390/molecules30153242 (PMC12348485; doi:10.3390/molecules30153242)
Supplement: Supplementary file 1 [file molecules-30-03242-s001.zip › molecules-3648993-supplementary figures.pdf]

## Supporting information for

# A Metabolomics Exploration of Young Lotus Seeds Using Matrix-Assisted Laser Desorption/Ionization Mass Spectrometry Imaging

**Ying Chen, Xiaomeng Xu, Chunping Tang**

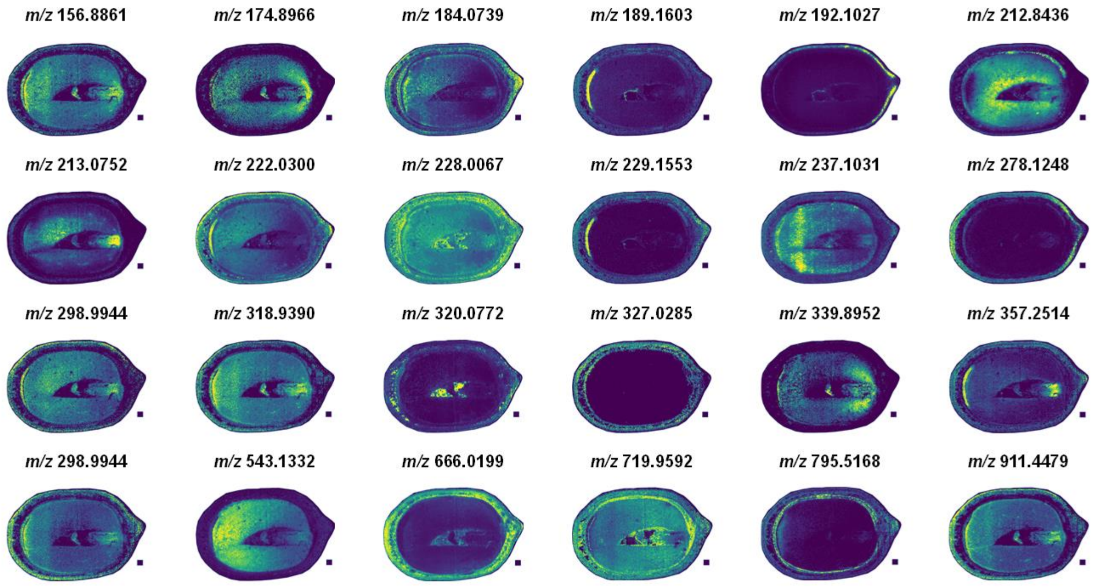


**Figure S1** Some images of the unidentified metabolites under positive ion mode detection.

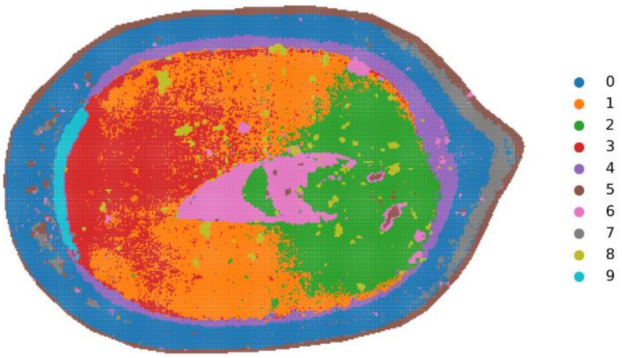


**Figure S2** The classification results of the MSI data (under positive ion mode detection) after K-means spatial clustering.
